# Integrative Therapy Use for Management of Side Effects and Toxicities Experienced by Pediatric Oncology Patients

**DOI:** 10.3390/children1030424

**Published:** 2014-11-14

**Authors:** Shana S Jacobs

**Affiliations:** Children’s National Medical Center, 111 Michigan Ave, NW, Washington DC 20010, USA; E-Mail: ssjacobs@childrensnational.org; Tel.: +1-202-476-2800

**Keywords:** complementary and alternative medicine, mind-body, traditional Chinese medicine, herbs, massage, acupuncture, yoga

## Abstract

Integrative Therapies (IT), otherwise known as Complementary and Alternative Medicine, are widely used among pediatric oncology patients, despite a paucity of available evidence. This review summarizes surveys that describe the prevalence of IT use by pediatric oncology patients, both during therapy and in survivorship, as well as the modalities being used. Additionally, the evidence that exists for specific treatments that appear to be efficacious in controlling specific symptoms is described. Finally, there are recommendations for practitioners on how to best counsel patients about IT use.

## 1. Introduction

Integrative therapies (IT), sometimes referred to as complementary and alternative medicine (CAM) refer to health care approaches with a history of use or origins outside of mainstream medicine, and incorporate a range of different health care approaches [1]. IT can be classified into mind-body interventions (hypnosis/hypnotherapy, meditation, faith healers, imagery), energy field therapies (acupuncture, acupressure, reiki, qi gong, therapeutic touch), body work (massage, osteopathic manipulation), and biological agents (vitamins, traditional Chinese medicine/herbs, nutritional supplements, plant extracts).

There are two compelling reasons for members of the health care team that treat pediatric oncology patients to increase familiarity with IT; first, IT is commonly employed by our patients and families and additionally, while the evidence for the effectiveness of these interventions is still building, some of these modalities are useful components of our supportive care arsenal for controlling the side effects of cancer treatments. In particular, the non-pharmacologic approaches are attractive given the possibility of ameliorating side effects without interacting with the many other medications patients are taking. This review will discuss both the types of IT being used by pediatric oncology patients, during therapy and in survivorship, and briefly mention those treatments that appear to be efficacious in controlling specific symptoms.

### 1.1. IT Use in Children and Adolescents with Cancer

Multiple studies have surveyed the prevalence of IT use among pediatric oncology patients. In 2010, Bishop *at al.* published a meta-analysis of all published surveys regarding IT use among pediatric cancer patients [2]. They found that these prior surveys yielded inconsistent results; IT prevalence ranged from 6%–91% in the prior studies (although most were in the range of 20%–60%). Some of the inconsistency resulted from different definitions of IT in different studies and different methodology. For example, some studies list types of IT in the survey, while others ask open-ended questions regarding use. In the latter case, some respondents might not consider their practices, such as taking vitamins, or spiritual healing, to be “IT” and therefore might under-report. Additionally, studies report on different patient populations, including multiple different countries, where practices may differ greatly. The most commonly and consistently reported IT modalities were herbal remedies, diets and nutrition, and faith-healing. IT was primarily employed by patients to cure or help fight the child’s cancer, to provide symptomatic relief, and to support conventional medicine, such as to relieve toxicities. The analysis reported that IT use was not associated with gender, age, ethnicity, or family income of pediatric patients with cancer.

Since that review was published there have been a few additional surveys of IT use in pediatric cancer. Gottschling recently published a population-based survey, covering 457 pediatric oncology patients across Germany and with a high response rate, suggesting that non-users were likely to be represented [3]. In this study, IT use actually dropped after the cancer diagnosis, with 41% reporting use prior to the diagnosis and only 31% since the diagnosis. Of note, this study did not include prayer/spiritual healing which may partly explain the low rates. An interesting finding in this study was that 78% of patients informed a physician of their IT use, a number higher than that cited in other studies, though only 16% of the attending pediatric oncologists reacted approvingly. Since a portion of patients stopped use after diagnosis, this may indicate that physician disapproval influences patients’ discontinuation of IT use. Other studies have documented that most patients and parents do not discuss their use of IT with their physicians, and most pediatric oncologists do not ask about IT use [4,5,6,7,8]. This fact is striking given that most pediatric oncology patients are participating in clinical trials where concomitant medications and their side effects are important to track and report [4], and further compounded as many patients increase IT use when upfront therapy fails [8], a time when many patients are enrolled in studies of investigational agents subject to even closer monitoring. In a study of adults enrolled in phase I trials, 52% of patients were using some sort of integrative therapy, and 77% of those were using a pharmacologic form of IT. Only 23% of patients revealed their IT use to their physician despite the importance of discussing all medications used during a phase I trial [9].

Interestingly, in Heath’s study of parents whose children died of cancer, parents who had open discussions with the treating physician about treatment alternatives were more likely to use IT [10]. This may suggest that physicians counseling patients at end of life might have been less negative about IT than those treating patients on cure-directed therapy.

Gottschling’s study also revealed differences in IT modalities used by different age groups; while both children and adolescents used homeopathy and massage most commonly, children used anthroposophic medicine (a practice very common in Germany but rare in the United States) third most while adolescents turned more to acupuncture and vitamin preparations. While the specific modalities may be most relevant for a German population, the concept that the usage varies by age is important to consider, perhaps reflecting different rates of acceptance of different modalities by younger *versus* older children, or different influences of parental experiences.

Multiple surveys report very high expectations of the patients using IT: In Gottschling’s study 68% of the children’s guardians and 55% of adolescents were confident or even absolutely sure that IT has beneficial effects. This is consistent with other studies where most patients report that IT therapies have helped them [7]. In Heath’s study of bereaved parents, the majority (78%) of respondents felt their child had benefited from IT use, even though parents whose children had used IT reported significantly lower levels of “child enjoyment” during the last month of life than parents whose children had not used IT [10].

## 2. Evidence for IT

### 2.1. Limitations of Research

Research on IT is complex and usually more difficult than that of conventional therapies, particularly in children with cancer. Preclinical and phase I studies are often not done. Traditional Chinese Medicine (TCM) and other traditional medicine techniques use a very personalized approach that individualizes treatment, using multiple herbs simultaneously, to restore an individual’s well-being rather than focusing more generally on a type of disease. The whole concept of disease and wellness is different than in Western medicine, and the herbs used have multiple components. These aspects of IT make good randomized clinical trials of the biological therapies very challenging [11,12] Investigations of non-pharmacologic therapies such as massage and acupuncture are hampered by the challenges of finding appropriate placebos, and the difficulty of ensuring a uniform approach between practitioners and from patient to patient [13,14]. Even in studies of adults with cancer, many of the studies done lack adequate controls, or are very small and underpowered to demonstrate efficacy [15,16]. The rarity of childhood cancer further reduces the incentive for many IT providers to scientifically evaluate therapies in children and adolescents and the ability of studies to show an effect of intervention.

### 2.2. Multiple Modalities

Despite the difficulties inherent in studying IT and the sometimes weak or conflicting evidence, several authors have tried to provide thorough reviews of the available evidence. Hunt evaluated IT use for all pediatric patients and concluded the evidence is strongest for acupuncture for post-operative nausea and vomiting, homeopathy for childhood diarrhea, massage for increasing sleep hours and hypnotherapy for reducing procedure-related pain [17]. Poder performed a systematic review of all non-pharmacologic forms of IT and found fair evidence for some modalities, including massage, acupuncture, imagery, art therapy, and music therapy. Because of faulty design methods, very few modalities were able to be rated more than “fair”, although evidence was slightly better for distraction and hypnosis which both have demonstrated improvements in pain, anxiety, distress, fear and nausea, especially in younger children. Evidence for chiropractic in pediatric oncology is lacking [18]. While evaluating non-pharmacological IT interventions specifically for pain and anxiety, Thrane found that most studies looked primarily at the effects of IT on these symptoms around procedures, and that hypnosis and music therapy were the most helpful in ameliorating procedure-related distress [19].

### 2.3. Massage

Massage is one of the more commonly employed forms of IT used by children in general and specifically children with cancer. In a review of massage in pediatric patients, Beider and Moyer reported on 24 randomized controlled trials, accounting for 200 participants, completed between 1992 and 2006. Despite the statistical and design flaws in many of the studies, the authors find strong evidence for the effect of massage on anxiety in children, particularly after repeated sessions of massage [20]. This finding is consistent with studies of massage in adults with cancer, where anxiety is the symptom most consistently reported as positively affected by massage, especially immediately after the massage [21,22,23,24]. Other symptoms improved by massage include pain, nausea, depression, anger, stress, and fatigue [25,26,27,28,29,30]. Most positive effects are short lived (immediately following the massage to within a half hour after), though some longer term effects have been noted [31,32].

In pediatric oncology patients, one small study of a mixed age, mixed diagnosis cohort revealed positive effects on reducing heart rate and anxiety, and participants had very positive evaluations of their massage experience [33]. Another Portuguese study revealed moderate improvements in pain interference [34]. A few studies have investigated massage in children undergoing bone marrow transplantation. In the first, 50 children with cancer undergoing bone marrow transplantation received professional massage, parent massage or were controls. Results indicated statistically significant differences in days to engraftment in the combined (parent and professional) massage group. Results from the professional massage group yielded a significant decrease in immediate anxiety and discomfort [35]. A follow up study at the same center compared a child intervention group (humor + massage), a parent intervention group (massage + relaxation) and standard of care control and found no differences in depression, quality of life, or post-traumatic stress, though all groups (intervention and control) improved over time and all groups had very high adjustment [36] Another smaller pilot study of three times weekly massage combined with acupressure *versus* standard of care control showed trends towards benefits in pain, nausea, fatigue and mucositits [37], and described high levels of parent/caregiver satisfaction with the intervention [38].

### 2.4. Acupuncture and Acupressure

Acupuncture describes a family of techniques in which small needles, heat, or electrical stimulation are placed at very precise anatomical points. Acupuncture is a key component of TCM. Acupuncture points are situated on meridians along which qi (a “life energy”) flows. Acupressure applies pressure (by hand or by another device) to the same acupuncture points.

In adult cancer patients, studies have demonstrated efficacy of acupuncture in managing symptoms such as nausea and vomiting [39,40,41], pain, fatigue, anxiety, and insomnia [42,43]. While evidence regarding use of acupuncture in pediatric conditions has not been as well demonstrated [14], acupuncture has been shown to be well tolerated in children [44,45], including no bleeding complications, even in thrombocytopenic patients [46]. Efficacy has been suggested in ameliorating pediatric pain (not oncology specific) [47,48,49] and in reducing chemotherapy-induced nausea and vomiting [50]. In addition, a meta-analysis of the effects of acustimulation (acupuncture, acupressure and electrical stimulation) on post-operative nausea and vomiting showed that acustimulation significantly reduced both vomiting and nausea [51].

Acupressure works similarly to acupuncture but uses pressure rather than needles, which is appealing for needle phobic children or when a trained practitioner is not available. The most common type of acupressure is the use of wrist bands which apply pressure to the P6 (Nei Kuan) acupressure point on the ventral surface of the wrist. In adults with cancer, a meta-analysis demonstrated that acupressure reduced both mean and worst acute nausea severity in conjunction with standard antiemetics [52,53]. However, a large 3-arm adult oncology study that incorporated a sham acupressure arm demonstrated a decrease in nausea in both the sham and the real acupressure arms, emphasizing the high potential for placebo effect in these types of studies [54]. A pilot cross-over study in pediatric oncology patients showed that acupressure is safe, feasible, and well-received [55]; a larger randomized study is ongoing.

### 2.5. Mind-Body Therapies

Landier *et al.* reviewed all studies of IT for procedure-related discomfort in pediatric oncology and found that several mind-body therapies, including hypnosis, cognitive distraction, and imagery, can be effective adjunctive therapies to manage procedure-related pain, anxiety, and distress. They found that hypnosis is particularly helpful for procedures highly associated with pain (e.g., bone marrows and lumbar punctures), and for reducing anticipatory anxiety, especially for children with higher levels of hypnotic susceptibility (often the 7–14 year age group) and when used in combination with pharmacological therapies [56]. Other reviews of non-pharmacological interventions for procedure-associated distress have also agreed that hypnosis was helpful [19,57]. Additionally, a small retrospective study of meditation in children with neuroblastoma receiving monoclonal antibody therapy showed that meditation significantly reduced analgesic use [58].

### 2.6. Energy Therapies

Energy healing therapy, or biofield therapies, “involves the channeling of healing energy through the hands of a practitioner into the client’s body to restore a normal energy balance and, therefore, health” [59]. Energy therapies include Reiki, therapeutic touch, and healing touch. These therapies have been minimally studied in adults with cancer. A recent review of biofield therapies in adults with cancer showed some conflicting effects on symptom reduction, but generally positive effects on pain reduction and psychological distress (anxiety, depression and stress), and improvements in quality of life. In several instances, the positive effects were not maintained, and there were some negative studies as well. In addition, most of the studies performed were not sufficiently powered or had a quasi-experimental design, limiting the usefulness of the data [60]. Another review of Reiki in adults with pain analyzed seven subjects (four were cancer patients) and suggested an effect on pain and anxiety [61]. There is even more limited data in pediatric oncology patients. One very small (nine patients) study of healing touch *versus* a “reading/play” control showed decreases in pain, stress, and fatigue for participants, parents, and caregivers [62]. Overall, energy therapies appear to be well received by children and adult cancer patients with no adverse effects, but the data supporting their use is lacking.

### 2.7. Yoga

Yoga aims to improve physical strength and flexibility as well as mental health through toning, stretching and relaxation training, and has been shown to affect the autonomic nervous system [63,64], reducing levels of salivary cortisol, plasma renin levels and urine nor-epinephrine and epinephrine levels [65], as well as lowering heart rate and blood pressure [66]. In adults with cancer, yoga has demonstrated improvements in fatigue [67,68]. Yoga has been found to be beneficial for physical and cardiopulmonary functioning in healthy children [69]. Two small studies showed that yoga is safe and feasible in pediatric cancer patients who are receiving chemotherapy [70,71], but further research is needed to demonstrate efficacy for symptom management.

### 2.8. Select Herbs and Biologic Therapies

Many herbs and biologic therapies are in use by patients with cancer and other disorders, and a full review of all potential biologic therapies is beyond the scope of this paper. Other reviews have examined some of these therapies in more detail [11,42,72,73,74]. Below are described select agents with evidence of effectiveness in symptom management or evidence of potential harm [75].

Traditional Chinese Medicine (TCM) refers to a health care practice approach that incorporates herbal medicines and various mind and body practices, such as acupuncture and tai chi, to prevent and treat health problems. Generally, several herbal medicines are combined by TCM practitioners and the herbs and amounts given are individualized to each patient [76]. Of note, there have been reports of contamination of Chinese herbs, with drugs, toxins, or heavy metals, or with inaccurate descriptions of ingredients, making these medicines particularly challenging not only to study but also to recommend for the pediatric oncology population. Some small studies have demonstrated efficacy of some components [77], for example SAMITAL, for prevention or treatment of mucositis in adults [76,78,79].

Melatonin is commonly used as a sleep aid, among other indications. A systematic review of randomized controlled trials of melatonin in conjunction with chemotherapy in adults with cancer showed some reductions in troubling symptoms (asthenia, leucopenia and thrombocytopenia, nausea and vomiting, and hypotension). There were no adverse effects noted, despite the fact that high doses were often used [80]. Melatonin has not been studied in children.

Ginger (*Zingiber officinale*) is often used as an antiemetic. It has been shown to be effective for nausea and vomiting associated with pregnancy [81,82,83,84], as well as shown promising, but inconsistent effects, on post-operative nausea and vomiting [85,86,87,88]. Several studies have assessed ginger for CINV in adults. In the largest study to date, Ryan *et al.* compared nausea severity in 644 adults receiving single-day chemotherapy who received one of three doses of ginger (0.5 g, 1.0 g, 1.5 g) to placebo. All doses of ginger significantly reduced nausea severity (*p* = 0.003), with the largest reduction in nausea severity at the 0.5 g/day and 1.0 g/day doses [89]. Ginger has not been well-studied in pediatric cancer patients.

Probiotics are one of the most frequently used biologic IT in children and adolescents. A number of studies have investigated *Lactobacillus rhamnosus GG* (Culturelle) in otherwise healthy children with viral-induced diarrhea and have reported encouraging findings. Because probiotics contain live active organisms, there has been hesitation in prescribing their use in immunocompromised patients. However, large, randomized, clinical trials in immunocompetent patients, including newborns, have rarely reported any adverse events [42]. Probiotics may be beneficial in the setting of allogeneic stem cell transplant. In a murine model of acute graft *versus* host disease (aGVHD), *L. rhamnosus GG* before and after transplantation resulted in improved survival and reduced Agvhd [90]. In a small study of children receiving chemotherapy, patients treated with the *Bifidobacterium breve* strain Yakult had fewer fevers and enhanced the habitation of anaerobes in the intestinal flora [91].

Glutamine is a conditionally essential amino acid during severe catabolic states and has been used for the prevention of mucositis and peripheral neuropathy. Although the optimal dosing and route of administration is not known, it appears promising in adult and pediatric oncology patients [92]. In studies of children undergoing stem cell transplants (SCT), glutamine decreased duration of fever [93] and use of both total parental nutrition (TPN) and narcotics compared to standard of care [94]. However, a small study of children receiving glutamine with chemotherapy showed no difference in the side effect profiles of the chemotherapy [95]. Glutamine has also been found to be helpful in preventing peripheral neuropathy in adults with cancer for example in patients on paclitaxel and oxaliplatin [96]. However, in a Children’s Oncology Group randomized controlled trial of glutamic acid for the prevention of vincristine toxicity in pediatric oncology patients, glutamic acid was not found to prevent neuropathy [97].

Carnitine deficiency has been noted in children and adolescents receiving chemotherapy, and has been found to correlate with fatigue [98]. Open-label studies in adult cancer patients, supplementation with L-Carnitine has been found to reduce cancer-related fatigue [99], however a large randomized trial showed no difference in fatigue compared to placebo [100].

Several herbal agents have been used for treating sleep disorders, a common complaint among cancer patients. Of these, Valerian *(Valeriana officinalis*) and Kava *(Piper methysticum)* have the most evidence in support. *Valerian* is a popular European herbal sedative. Multiple studies have shown Valerian to improve sleep without increasing daytime sleepiness. Additionally, it appears to have a wide margin of safety and it is not metabolized via the cytochrome enzymes, suggesting no interaction with chemotherapy agents. However, it has not been well studied in cancer patients or in children, and effects of long-term use are unknown. *Kava* also appears to be efficacious and is generally well tolerated, but there have been a few reports of hepatic failure associated with *Kava* use, making it not advisable to use concurrently with chemotherapy [101].

Antioxidants (such as beta-carotene, lycopene, Vitamins C, E, and A, and other substances) are another common class of supplements used by patients with cancer. In a prospective observational study conducted among children with Acute Lymphoblastic Leukemia (ALL), low plasma and dietary antioxidant levels directly correlated with treatment related toxicity [102]. However, there is concern that supplements promoted for their antioxidant qualities may diminish the efficacy of anticancer treatments, particularly radiation, that work through promotion of oxidizing free radicals [11,103]. Studies adequately evaluating the impact of supplementation on toxicity and disease free survival have not been completed, making it difficult to recommend these supplements for oncology patients on therapy.

Milk thistle, a hepatoprotective herb, is associated with reductions in serum transaminases in children receiving maintenance chemotherapy for ALL [104]. Its use may reduce the need for dose reductions in chemotherapy for hepatotoxicity, but larger randomized studies are warranted.

Fish oil, or omega-3 fatty acids (eicosapentaenoic acid (EPA) and docosahexaenoic acid (DHA)) are naturally found in fish and are thought to protect against inflammation. Supplementation with fish oils have been shown to have some benefit for adults undergoing chemotherapy in terms of weight gain and quality of life [105,106] and is often used in children with cognitive and attention disorders, conditions which mimic some of the long term cognitive deficits in survivors of childhood cancer, though the evidence is still in development [107,108]. Since high doses of these supplements are well tolerated for various conditions, there appears little down side to recommending fish oil supplementation for childhood survivors experiencing or at risk of cognitive problems.

## 3. Risks of IT

Integration of IT into pediatric oncology, even as an adjunct for symptom management, is controversial. The evidence supporting the safety and efficacy of herbal and biologic treatments alongside or even following chemotherapy is lacking, the herbs are not regulated by the Food and Drug Administration, and systematic reporting is not legally mandated [72]. As mentioned above, there have been reports of heavy metal and microbial contamination of TCM herbs [109]. On the other hand, in a summary of all adverse events associated with the use of IT in children reported to an Australian registry, most of the severe events (and all of the death) were connected to those patients who used IT in lieu of conventional medicine, as opposed to as an adjunct [110]. While many side effects of herbs and interactions with chemotherapy remain unknown, some of the known and theoretical risks and interactions are available [73,111,112].

The non-biologically based therapies are generally considered safe and there is a low risk of interference with conventional therapies. However, training programs for IT practitioners often have little to no standards for training with children, let alone children receiving intensive conventional treatment. Furthermore, most pediatric hematology/oncology fellowship curricula do not have formal educational on IT; therefore, pediatric oncologists have little information and are ill-prepared to advise patients on how to safely incorporate IT into their treatment.

## 4. How to Best Counsel patients about IT use

Given the inadequate quantity and quality of research on integrating IT into standard chemotherapy treatment, many practitioners simply advise against IT use. However, the popularity of IT, and the frequent under-reporting of IT use among cancer patients and their families suggests that it is necessary to be open to discussing and even recommending certain forms of IT. In general, since non-pharmacological forms of IT have not been found to be harmful—some studies have suggested benefits—and many patients are eager to try alternatives to medications for symptom relief, it makes sense for oncologists to encourage patients to try these modalities when appropriate, available, and not cost-prohibitive. In terms of the biological therapies, it is important to ask patients if they are taking any types of herbs or supplements, or if they plan to. While they must always be cautioned about the potential risks as discussed above, the specific supplements they are interested in can be further researched for known toxicities or interactions to make an informed decision. Some reputable sites with comprehensive summaries of known complementary treatments include the Society of Integrative Oncology Guidelines [113], and National Center for Complementary and Alternative Medicine “Topics A to Z” [114]. See Table 1 for a summary of IT modalities, associated symptoms and evidence for use.

**Table 1:** Integrative Therapy (IT) modalities, relevant symptoms, and evidence in support.

Modality	Symptoms used for	Comments
Massage	Anxiety, pain, nausea, depression, anger, stress, and fatigue, discomfort, mucositis	Most evidence for reduction of anxiety, especially in adults with cancer; some evidence in pediatric oncology
Acupressure and acupuncture	Nausea and vomiting, pain, fatigue, anxiety and insomnia	Specific evidence for chemo-induced nausea and vomiting in pediatric oncology, and pediatric pain (non-cancer specific)
Mind-body therapies, including hypnosis, cognitive distraction, meditation and imagery	Procedure-related pain, anxiety, and distress	Evidence in pediatrics, especially for hypnosis
Energy therapies, or biofield therapies (healing touch, therapeutic touch, Reiki)	Pain, anxiety/depression/stress, fatigue	Scant evidence in adults and children
Yoga	Fatigue, stress	Scant evidence in pediatric oncology
Traditional Chinese Medicine	All symptoms	Very limited evidence in pediatric oncology
Melatonin	Asthenia, leucopenia and thrombocytopenia, nausea and vomiting, and hypotension	Not studied in children
Ginger	Nausea	Evidence in adult oncology
Probiotics	Diarrhea, graft vs host disease	Minimal evidence in pediatric oncology
Glutamine	Peripheral neuropathy and mucositis	Mixed evidence for mucositis in pediatric oncology; poor evidence for use in neuropathy
Carnitine	Fatigue	Not studied in pediatrics; mixed results in adult oncology
Valerian	Sleep problems	Not well studied in pediatrics or cancer patients
Kava	Sleep problems	Risk of liver toxicity limits use
Milk thistle	Liver toxicity	Limited evidence in pediatric ALL
Fish oil	Malnutrition, cognitive disorders	Evidence in adults with cancer and children with ADHD

## 5. Summary

The goal of an integrative approach in the care of children and adolescents with malignancies is to provide IT modalities that are deemed safe and effective in conjunction with effective conventional medical treatments. There is significant need for research evaluating the roles of IT for symptom control of toxicities related to conventional therapies. More formal education opportunities on IT in the training of pediatric oncologists and other healthcare providers working with children and adolescents with cancer should be implemented. Healthcare providers should consider how IT services may be beneficial to children and adolescents with cancer. However, until the evidence for or against an integrative modality is more conclusive, the provider’s role is to ask about and document the use of IT, critically evaluate the evidence or lack of evidence, balance the potential risks with possible benefits, and assist the family in their decisions regarding use of integrative approaches for their child.
